# Clinical efficacy and safety of Xuefu Zhuyu decoction in the treatment of diabetic kidney disease: A protocol for systematic review and meta-analysis

**DOI:** 10.1097/MD.0000000000032359

**Published:** 2022-12-23

**Authors:** Chaoqun Song, Zhiyue Zhu, Miao Liu, Wenbo Yang, Xiaotian Bai, Zheng Nan

**Affiliations:** a College of Chinese Medicine, Changchun University of Chinese Medicine, Changchun, China; b Department of Pediatrics, Affiliated Hospital of Jiangxi University of Chinese Medicine, Nanchang, China; c Zhangjiakou Institute of Traditional Chinese Medicine, Zhangjiakou, China.

**Keywords:** DKD, meta-analysis, protocol, Xuefu Zhuyu decoction

## Abstract

Diabetic kidney disease (DKD) is one of the common complications of diabetes, the most crucial cause of end-stage renal disease, and a significant cause of death in diabetes patients. In recent years, Xuefu Zhuyu decoction has also been widely used in treating diabetic nephropathy. However, there need to be more systematic reviews that comprehensively describe the effectiveness and safety of Xuefu Zhuyu decoction in treating DKD. Therefore, we will conduct a systematic review and meta-analysis to evaluate the indicators of Xuefu Zhuyu decoction in the treatment of DKD to provide evidence-based support.

**Methods:**

Eligible published randomized controlled trials from January 2005 to October 2022 will be obtained by searching PubMed, Cochrane Library, EMBASE, Web of Science, CNKI, Wanfang, and VIP in Chinese and English. The Cochrane Collaboration Risk of bias tool will be used for methodological quality assessment and risk of bias. The meta-analysis will be performed using Cochrane RevMan 5.4 software.

**Results:**

This study will compare the following indicators: the primary outcomes: urinary albumin excretion rate and urea nitrogen. Secondary outcomes: blood creatinine; 24 hours urine protein quantification; glycosylated hemoglobin; fasting blood glucose; 2-hour postprandial blood glucose; total cholesterol; triglycerides; total effective rate; incidence of adverse events.

**Conclusion:**

The results of this systematic review will provide an objective, evidence-based basis for judging the efficacy and safety of Xuefu Zhuyu decoction in treating DKD.

## 1. Introduction

Diabetic kidney disease (DKD) is one of the common complications of diabetes, the leading cause of end-stage renal disease, and a major cause of death in patients with diabetes.^[1]^ According to the International Diabetes Federation (IDF), there were 415 million adult diabetes patients worldwide as of 2015, and this is predicted to rise to 642 million by 2040.^[2]^ The exact mechanism of DKD is uncertain, with hyperglycemia, hypertension, and genetic predisposition being the main risk factors.^[3]^ Although strict control of blood pressure, blood glucose, and suppression of renin-angiotensin-aldosterone may confer benefits in DKD, it does not significantly reduce morbidity and mortality.^[4]^ Traditional Chinese medicine believes that blood stasis obstruction is an important mechanism and runs through the whole course of DKD, so the treatment of this disease must adopt the method of activating blood and removing stasis. Xuefu Zhuyu decoction is known as the first party to activate blood and remove stasis from Wang Qingren’s “Yilin Gaicuo,” which is an active prescription that is widely used to improve blood circulation by removing blood stasis.^[5]^ It is composed of 11 herbs: angelica, raw Rehmannia, peach kernel, safflower, citrus husk, red peony, chai hu, licorice, bellflower, Chuanxiong, hyssop, with the effect of activating blood and removing stasis, practicing qi and relieving pain.^[6]^ Several studies have shown that Xuefu Zhuyu decoction has the effects of inhibiting platelet aggregation, improving blood rheology and microcirculation, alleviating clinical symptoms and so on.^[7–10]^ Xuefu Zhuyu decoction is often used to treat cardiovascular and cerebrovascular system diseases, tumor and so on.^[11,12]^ In recent years, Xuefu Zhuyu decoction has also been widely used in the treatment of DKD. This study will comprehensively collected the randomized controlled trials (RCTs) of Xuefu Zhuyu decoction alone or combined with hypoglycemic drugs to treat DKD and evaluate the clinical efficacy and safety of Xuefu Zhuyu decoction, in order to provide a reference for follow-up research and clinical practice of DKD.

## 2. Method

### 2.1. Agreement registration

This study is registered with PROSPERO, registration number: CRD42022373832 (https://www.crd.york.ac.uk/PROSPERO/display_record.php?RecordID=373832). The protocol will be reported according to the preferred reporting items of the Systematic Review and Meta-Analysis Protocol (PRISMA-P)^[13]^ and the Cochrane Intervention System Evaluation Manual.^[14]^

### 2.2. Inclusion criteria

The group of study participants will be comprised of adults diagnosed with DKD as based on clear clinical diagnostic criteria, regardless of the course of the disease, sex, age, race, nationality, education, or financial status.

#### 2.2.1. Types of studies.

The type of study will only include RCTs of Xuefu Zhuyu decoction for DKD. Animal experiments, mechanistic studies, retrospective analyses, cohort studies, case reports, data analysis, clinical guidelines, experiences, reviews, conference papers, or dissertations will be excluded.

#### 2.2.2. Participant characteristics.

The selected patients need to conform 1999 WHO diagnostic criteria for diabetes, the Mogensen criteria for staging DKD, and excluded non-diabetic renal impairment.^[15]^

#### 2.2.3. Interventions.

Interventions involving Xuefu Zhuyu decoction or modified Xuefu Zhuyu decoction will be included. They will not be limited to the dosage forms (decoction, capsule, or granules), frequency, or dose, but the method of administration will be limited to oral administration. The experimental group can be Xuefu Zhuyu decoction alone or Xuefu Zhuyu decoction combined with conventional treatment, and no other TCM treatment is involved.

#### 2.2.4. Control measures.

The control group can be treated with a placebo or conventional treatment. Conventional treatment will include diabetes health education, diet management, exercise intervention, blood glucose monitoring, and hypoglycemic drug treatment. There will be no restrictions on the types and dosage forms (oral, injection) of hypoglycemic drugs. The experimental group should be the same as the control group if combined with conventional treatment.

#### 2.2.5. Results.

Primary outcomes: urinary albumin excretion rate and urea nitrogen.

Secondary outcomes: blood creatinine, 24 hours urine protein quantification, glycated hemoglobin, fasting blood glucose, 2-hour postprandial blood glucose, total cholesterol, triglycerides, total clinical efficiency and incidence of adverse events.

#### 2.2.6. Exclusion criteria.

The following literature will be excluded: non-randomized controlled trials and literature without full text; literature that does not conform to diagnostic criteria; literature published repeatedly; kidney damage not caused by diabetes; research on non-oral Xuefu Zhuyu decoction; including other TCM laws; lack of data or incomplete data; research on dialysis patients.

### 2.3. Information sources and retrieval strategies

We will search from the following 7 databases: PubMed, Cochrane Library, EMBASE, Web of Science, China National Knowledge Infrastructure (CNKI), Wanfang, and China Science Journal Database (VIP), from January 2005 to October 2022. The clinical trials related to oral Xuefu Zhuyu decoction, modified Xuefu Zhuyu decoctions, DKD will be searched using a combination of subject terms and text words.The search terms mainly included: “Xuefuzhuyu decoction,” “Xuefuzhuyu,” “Xuefuzhuyu Tang,” “Xuefu Zhuyu decoction,” “Xuefu Zhuyu Tang,” “XFZY,” “diabetic kidney disease,” “diabetic kidney diseases,” “diabetic nephropathy” and “randomized controlled trials.” In addition, if possible, standard search terms from RCTs will be used. The search strategy has no planning restrictions to prevent overlooking essential studies that are not classified correctly in their respective bibliographic databases. Table 1 summarizes the PubMed search strategies.

**Table 1:** Search strategy in English and Chinese databases.

Number	Keywords of the research
#1	Diabetic kidney disease [mh] OR Diabetic nephropathy [mh] OR Diabetic nephropathies [mh]
#2	Diabetic nephropathy*[tiab] OR Diabetic kidney disease*[tiab] OR Diabetic kidney lesion [tiab] OR Diabetic kidney damage [tiab] OR Diabetic renal disease [tiab] OR Diabetic renal lesion [tiab] OR Diabetic renal damage[tiab]OR renal dysfunction [tiab] OR DKD [tiab] OR CKD [tiab]
#3	#1 OR #2
#4	Xuefuzhuyu decoction [tiab] OR Xuefuzhuyu [tiab] OR Xuefuzhuyu Tang [tiab] OR Xuefu Zhuyu decoction [tiab] OR Xuefu Zhuyu Tang [tiab] OR XFZY [tiab]
#5	Randomized controlled trial [pt] OR Controlled clinical trial [pt]
#6	Randomized controlled trial [tiab] OR Controlled clinical trial [tiab] OR Randomized* [tiab] OR Randomly* [tiab] Random allocation [tiab] OR Trial [tiab] OR CCT [tiab] OR RCT [tiab]
#7	#5 OR #6
#8	#3 AND #4 AND #7

DKD = diabetic kidney disease, mh = MeSH, tiab = tittle/abstract, RCT = randomized controlled trial.

### 2.4. Data collection and analysis

#### 2.4.1. Study selection.

**Figure 1.**
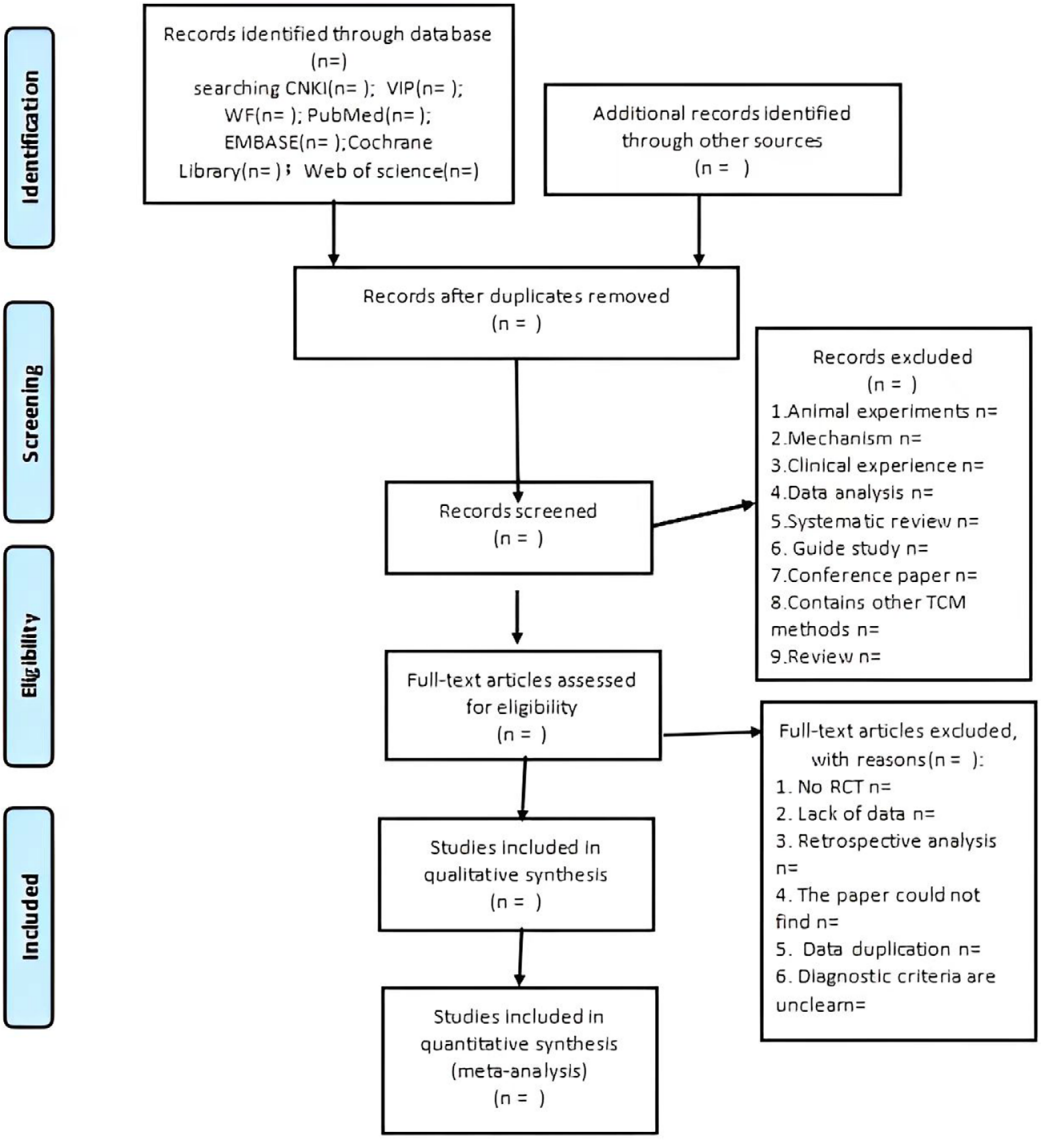
PRISMA flow diagram of study and exclusion. PRISMA = preferred reporting items for systematic reviews and meta analyses.

Before selecting studies, all reviewers will be professionally trained to understand the objectives and process of the review. We will conduct screening studies using reference management software (EndNoteX9). First, duplicate literature will be eliminated. Secondly, non-compliant literature will be carried out by reading the title and abstract. Then, we will reading the entire article to exclude articles that do not meet the inclusion criteria. Throughout the process, 2 authors will extract data independently and mediate by an independent author for cases with disagreement. Any disagreements will be resolved through discussion until consensus is reached or a third author is consulted. The flowchart of the screening process is shown in Figure 1.

#### 2.4.2. Extraction of data.

The data items of eligible studies will be extracted independently by 2 reviewers. Relevant data will be entered into Microsoft Excel 2016 tables, including source, 1^st^ author, year of publication, study design, diagnostic criteria, randomization method, blinding, sample size, sex, mean age, duration of disease, duration of treatment, interventions, outcomes, adverse events, et cetera. And the data will be cross-checked by a third investigator to ensure quality. In case of disagreement, a third party will resolve it through discussion or mediation.

#### 2.4.3. Missing data management.

We will contact the original author of the paper with missing data by email or phone and wait for a response from the author after contact. If missing data cannot be obtained, papers containing incomplete data will be excluded from the final analysis. Studies will be excluded if full texts were unavailable through an online search or by contacting authors.

#### 2.4.4. Risk of bias.

Reference quality will be assessed using the risk of bias risk assessment tool in the Cochrane Handbook. The tool will assess 7 critical sources of bias, including random sequence generation, allocation concealment, blinding of participants and personnel, blinding of outcome assessment, incomplete outcome data, selective reporting, and other biases. By assessing the completeness of the study and the correctness of the methodological implementation, each aspect will be assessed as “high risk,” “low risk,” or “unclear risk.” The 2 researchers will perform independently and examine each other. If there is a disagreement on the assessment results, the third researcher will participate in the discussion and make a final decision.

### 2.5. Statistical analysis

#### 2.5.1. Heterogeneity tests and meta-analyses.

RevMan 5.4 will be used for meta-analysis. For binary variables, the effect size will be expressed using relative risk (RR) and 95% confidence interval (CI). For continuous variables, mean difference (MD) and 95% CI will be used to indicate effect size when the same unit is used for the same outcome measure. Otherwise, standardized mean difference (SMD) and 95% CI will be used. The *χ*^2^test and the inconsistency index (*I*^2^) test will be used to evaluate heterogeneity. If *P* > .1 and *I*^2^ < 50%, this indicates that heterogeneity between studies is small, and a fixed-effect model will be used to calculate the mixed effect size. If *P* ≤ .1 and/or *I*^2^ ≥ 50%, it indicates significant statistical heterogeneity between studies; therefore, a random-effects model will be used. At the same time, subgroup analysis and sensitivity analysis will be used to explore the sources of heterogeneity and judge the stability of the study results. Subgroup analysis will be used as needed to group different influences, such as different stage of DKD or different drug dosage of Xuefu Zhuyu decoction, while sensitivity analysis will be used to help screen out studies with high risks of bias.

#### 2.5.2. Sensitivity analysis.

For the stability of the findings, we will use the Stata software to perform a sensitivity analysis of the results. When significant statistical heterogeneity was found, we will exclude low-quality trials, duplicate meta-analysis papers, and studies with insufficient sample size and insufficient data included in the analysis. Then, we will re-analyze and pool the data and compare the differences between the regained and original effects. In this way, we will assess the impact of individual studies on the overall results and whether the results are reliable.

#### 2.5.3. Evaluation of publication bias.

Publication bias will be visually checked graphically using a funnel plot and tested using Egger’s test, with approximate symmetry indicating no publication bias.

#### 2.5.4. Subgroup analysis.

In order to obtain reliable data, subgroup analyses will be performed based on the duration of the intervention, different stage of DKD and several different drug dosages of Xuefu Zhuyu decoction.

#### 2.5.5. Recommended classification of evidence quality.

The certainty of the evidence for meta-analyses related to patient outcomes will be assessed using the criteria for Assessment, Development, and Evaluation of Recommendations (GRADE) developed by WHO and international organizations. We will judge the certainty of the evidence based on the level of evidence for the results of RCTs based on methodological quality, consistency of findings, directness, and accuracy of evidence, and the likelihood of publication bias. The GRADE system will classifiy the quality of the evidence into 4 levels: high, moderate, low, and very low.

### 2.6. Ethics and communication

Studies included in our analysis will have been published publicly and thus will not require ethical approval for inclusion in the study. Protocols for this systematic review will be published in peer-reviewed journals and presented at relevant national and international conferences.

## 3. Discussions

Currently, there is no specific drug for treating DKD. However, Traditional Chinese medicine has a significant effect on the treatment of chronic kidney disease, and some experts suggest that using triptolide and emodin can improve the treatment of proteinuria in the treatment of chronic kidney disease,^[16,17]^ traditional Chinese medicine ingredients such as ginsenosides, tanshinone IIA, anthocyanin also have beneficial effects on DKD.^[18–20]^ Xuefu Zhuyu decoction, as the first prescription of promoting blood circulation and removing blood stasis, it has more clinical RCTs for DKD, especially in China. However, there is no systematic review comprehensively describing the effectiveness and safety of Xuefu Zhuyu decoction for DKD. Therefore, we plan to conducte a systematic review to evaluate the indicators of Xuefu Zhuyu decoction in treating DKD to provide convincing evidence of the efficacy and safety of it. In this systematic review, we will make every effort to improve the reliability of the results, including but not limited to systematic literature searches, rigorous risk of bias, reasonable data analysis, objective assessment of the quality of evidence, and rigorous protocol-based studies. We believe that the results of this systematic review will provide the best available evidence to demonstrate the efficacy and safety of Xuefu decoction in treating DKD.

## Acknowledgements

The authors thank the National key research and development program of China for financial support.

## Author contributions

All authors contributed to the drafting of the final protocol.

**Conceptualization:** Chaoqun Song.

**Data curation:** Miao Liu.

**Formal analysis:** Miao Liu.

**Project administration:** Zheng Nan.

**Supervision:** Zhiyue Zhu, Wenbo Yang, Xiaotian Bai.

**Writing – original draft:** Chaoqun Song.

**Writing – review & editing:** Wenbo Yang, Zheng Nan.
